# Ongoing behavior predicts perceptual report of interval duration

**DOI:** 10.3389/fnbot.2014.00010

**Published:** 2014-03-11

**Authors:** Thiago S. Gouvêa, Tiago Monteiro, Sofia Soares, Bassam V. Atallah, Joseph J. Paton

**Affiliations:** Champalimaud Neuroscience Programme, Champalimaud Centre for the UnknownLisbon, Portugal

**Keywords:** interval timing, time perception, decision making, perceptual decision making, choice probability, embodied cognition

## Abstract

The ability to estimate the passage of time is essential for adaptive behavior in complex environments. Yet, it is not known how the brain encodes time over the durations necessary to explain animal behavior. Under temporally structured reinforcement schedules, animals tend to develop temporally structured behavior, and interval timing has been suggested to be accomplished by learning sequences of behavioral states. If this is true, trial to trial fluctuations in behavioral sequences should be predictive of fluctuations in time estimation. We trained rodents in an duration categorization task while continuously monitoring their behavior with a high speed camera. Animals developed highly reproducible behavioral sequences during the interval being timed. Moreover, those sequences were often predictive of perceptual report from early in the trial, providing support to the idea that animals may use learned behavioral patterns to estimate the duration of time intervals. To better resolve the issue, we propose that continuous and simultaneous behavioral and neural monitoring will enable identification of neural activity related to time perception that is not explained by ongoing behavior.

## 1. Introduction

Animals live in naturally stochastic environments where apprehending environmental regularities is extremely important. In particular, being able to identify temporal regularities in the environment enables animals to predict future events such as the presence of mates, food or danger (Balsam and Gallistel, 2009), or to decide between alternative courses of action, e.g., deciding when to switch from exploiting a depleting food patch to exploring for new ones so as to optimize energy balance (Kacelnik and Brunner, 2002; Bateson, 2003). Behaviorally relevant temporal regularities in the environment are often on the scale of multiple seconds, therefore understanding how organisms handle time durations on this scale is extremely important to understand behavior itself.

Traditional sensory modalities such as vision, audition or tactile sensation are processed by known sensory organs and brain areas. Time perception, on the other hand, still lacks a clear and direct demonstration of how it would be implemented within the nervous system. In addition, whether the representation of temporal information is localized or distributed across different brain areas is still a matter of debate and ongoing research (Ivry and Spencer, 2004).

Neurally inspired models for interval timing include those that involve coincidence detection among oscillations of varying frequencies (Miall, 1989; Matell and Meck, 2000, 2004), integration of the noisy firing of neural populations (Simen et al., 2011) and variable firing dynamics within a population of neurons (Grossberg and Schmajuk, 1989; Buonomano and Merzenich, 1995; Meck et al., 2008; Shinomoto et al., 2011) as encoding schemes for time related information. Additionally, several abstract models of how animals track the passage of time have been proposed, many of which fall in one of two categories: accumulator models tell time by counting pulses emitted by a pacemaker and comparing it to a remembered value (Gibbon, 1977), while state based models represent time as a trajectory progressing through a sequence of states (Killeen and Fetterman, 1988; Machado, 1997; Ludvig et al., 2008). A subset of sequential state timing models posit that states reflect behavior (Killeen and Fetterman, 1988; Machado, 1997), stemming from the widely replicated observation that structured behavioral chains emerge under temporally structured reinforcement contingencies (e.g., Skinner, 1948; Hodos et al., 1962; Anderson and Shettleworth, 1977; Haight and Killeen, 1991; Machado and Keen, 2003; Balcı et al., 2008; Ölveczky, 2011; for reviews, see Staddon and Simmelhag, 1971; and Staddon, 1977).

Suggesting that interval timing is driven by behavioral state transitions implies a clear prediction that, to our knowledge, has not yet been tested: variation of behavioral chains should correlate with variations in time estimation. In the current work we continuously monitored the behavior of rodents as they categorized interval durations as longer or shorter than a learned standard interval. Idiosyncratic behavioral sequences displayed during the interval being timed were highly reproducible across trials and sessions. Moreover, the small variation present in the behavioral trajectories was often predictive of temporal judgments from very early in the trial, sometimes in advance of trial onset. These results revealed a correlation between learned motor behavior and perceptual report of elapsed time.

## 2. Results

### 2.1. Animals learned to categorize time intervals

**Figure 1:**
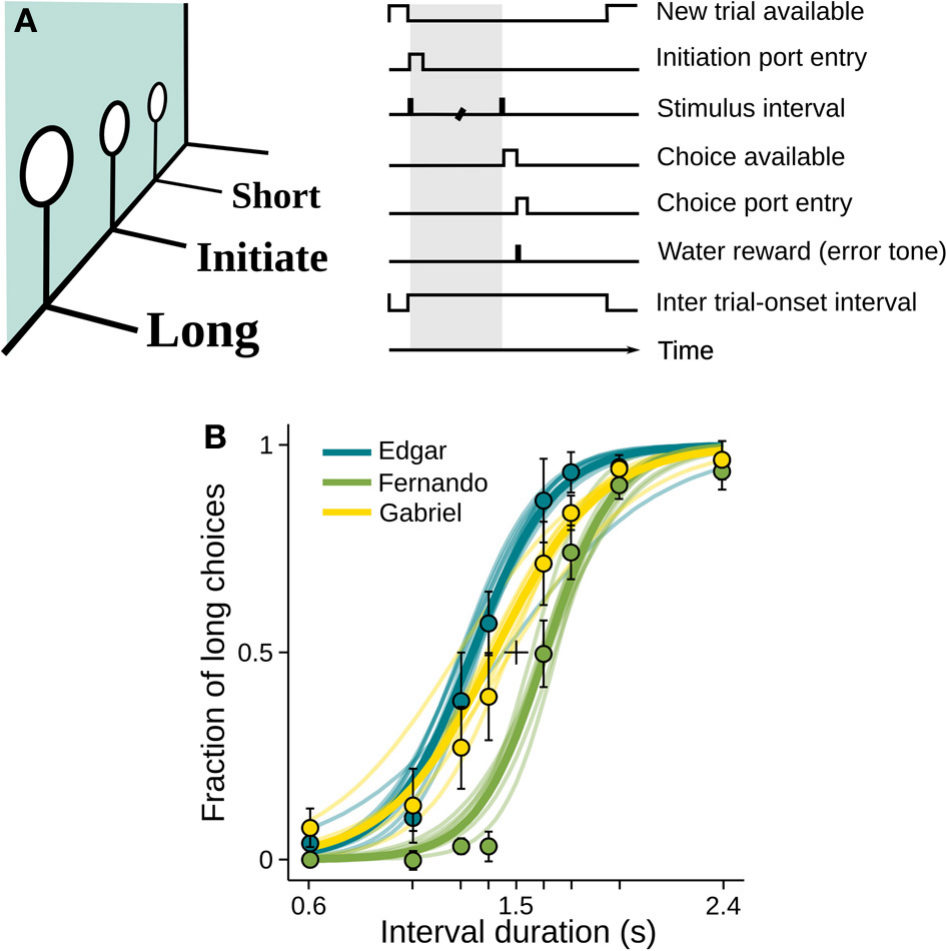
**Rats learned to categorize interval durations as short or long**. **(A)** Animals interacted with three nose ports. Poking at the central port triggered a random stimulus interval (range: 0.6–2.4 s; top right, shaded area). A water reward became available at the left port after intervals longer than 1.5 s, and at the right port after shorter intervals. Wrong choices were punished with a brief white noise sound and a time out. **(B)** Psychometric functions show near perfect categorization of easiest stimuli (i.e., those far from the 1.5 s categorical boundary), while performance approaches chance level for intermediate durations. Circles and whiskers are mean and standard deviation across sessions. Thin lines are logistic fits to single sessions. Thick lines summarize performance of each subject. Color code identify subjects and is maintained throughout the article. *n* = {10, 8, 6} sessions (rats Edgar, Fernando, and Gabriel, respectively; same for Figures 3, 5, 6); 346 ≤ *n* ≤ 558 trials per session.

We trained three rats and one mouse to categorize time intervals as either long or short by making left/right choices (Figure 1A and Materials and Methods). At each self initiated trial, two brief tones were played separated in time by an interval randomly selected from the set *I* = {0.6, 1.05, 1.26, 1.38, 1.62, 1.74, 1.95, 2.4} s. Judgments about interval duration were reported at two laterally located nose ports: choosing the left side was reinforced with a drop of water after intervals longer than 1.5 s, and the right side otherwise. Incorrect choices were punished with an error tone and a time out. Animals were free to move during stimulus presentation, as long as they withheld choice until interval offset.

Each interval presentation followed by a choice constitutes one trial, and rats performed on average 456 trials per daily session (minimum = 346, standard deviation = 49.4). As revealed by their psychometric functions, animals made virtually no errors when categorizing the easiest (i.e., shortest and longest) intervals, but categorization performance declined as intervals came closer to the 1.5 s categorical boundary (Figures 1B, S1A).

### 2.2. Animals developed temporally structured behavior

**Figure 2:**
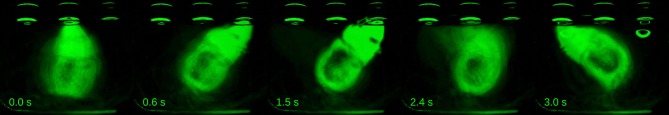
**Behavior displayed during stimulus interval is highly reproducible**. A series of video frames taken from a representative session of rat Fernando at specific time points within trials were averaged across all presentations of the longest interval (I = 2.4 s). Times when frames were taken are indicated in seconds relative to interval onset. *n* = 62 trials.

Apart from the discrete, temporally sparse behavioral measurements obtained from the nose ports, we monitored behavior continuously with a high-speed camera (rats: 120 fps; mouse: 90 fps; see Materials and Methods for details). The camera was located at the top of the behavioral box, thus only detecting motion occurring within planes parallel to the floor. Videos taken around presentation of stimulus intervals revealed highly consistent body motion patterns. To illustrate this, we overlaid video excerpts of a representative session of rat Fernando time-aligned at the onset of presentations of the longest interval. This interval duration was chosen for the analysis because it allows behavioral sequences to unfold for as long as possible before being disrupted by the interval offset tone, which acts as a go signal prompting the animal to move to a choice port. As a result of the high degree of reproducibility of the behavioral sequence, the resulting averaged images are surprisingly sharp (Figure 2, Supplementary Movie 1).

**Figure 3:**
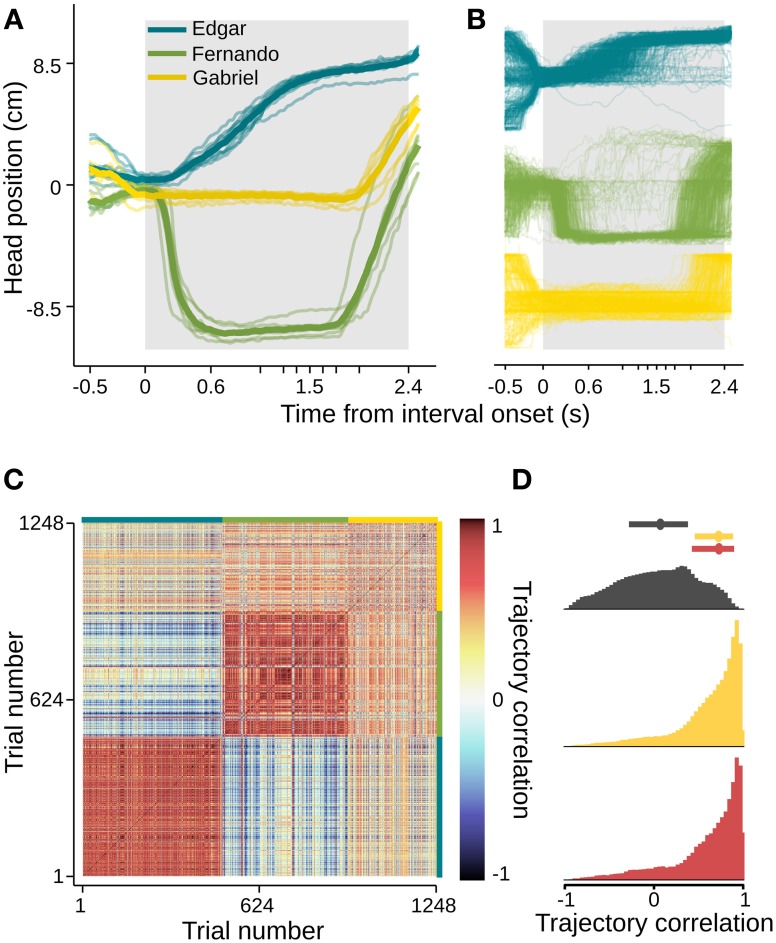
**Head trajectories are reproducible and idiosyncratic**. **(A)** Head trajectories around presentations of longest interval. Thin lines are single session means. Thick lines are means across session means. **(B)** Trajectories at all single trials when the longest interval was presented. **(C)** Matrix of pairwise correlations between trajectories shown in **(B)**. Trials are ordered by subject (color bars framing top and right margins), then session, then trial number. **(D)** Normalized histograms of correlation coefficients. Trajectories occurring in the same session are strongly and positively correlated (bottom, red). The same is true for pairs of trajectories occurring at different sessions of a same subject (middle, yellow), and the two distributions are indistinguishable (Kolmogorov–Smirnov test, *p* = 0.84). Trajectories of different subjects, however, show near-zero correlations (top, dark gray). The latter distribution differs significantly from both within subject coefficient distributions (Kolmogorov–Smirnov tests, *p* < 0.01 in both cases). Dots and bars above histograms are medians and interquartile ranges. *n* = {10, 8, 6} sessions; *n* = {491, 445, 312} trials.

In order to quantify this effect and extend our analysis to a number of subjects and sessions, we used the aid of computer vision tracking algorithms to follow the position of each animal's head in time (see Materials and Methods for details). In agreement with the example video, head trajectories revealed body motion patterns that were very consistent across trials, as well as across sessions (Figures 3A,B, S1). Interestingly, each subject developed its own distinct trajectory. To assess the within and between subject variability in head trajectory, we computed a correlation matrix comparing all pairwise combinations of trials wherein the longest interval duration was delivered. Correlations between trajectories produced by a given subject were highly and consistently positive, whether or not they occurred in the same session. Pairs of trajectories of different subjects, on the other hand, showed near zero correlations (Figures 3C,D). Given the observation that trajectories are idiosyncratic and consistent from session to session, we pooled data across sessions within subjects for the following analyses.

### 2.3. Ongoing behavior bears information about unfolding perceptual decisions

**Figure 4:**
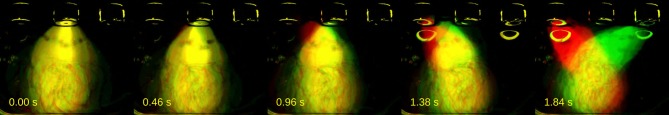
**Distinct behaviors accompany different categorizations of same stimulus**. A series of video frames were taken from a representative session of rat Edgar at specific time points during presentations of a near-boundary stimulus interval (*I* = 1.38 s). Short choice trials were put on the green, and long choice trials on the red channel. Times when frames were taken are indicated in seconds relative to interval onset.

Next, we asked whether trial-to-trial variability in body trajectories carried information about the perceptual decisions being forged; were this the case, different categorizations of the same stimuli should be accompanied by distinct behavioral trajectories. For a representative session of rat Edgar, we selected all presentations of the stimulus for which choice variance (σ^2^_choice_) was highest (*I* = 1.38 s; *n* = 56 trials; σ^2^_choice_ = 13.3571). The different color channels in the video were used to parse trials by choice: long choice trials were put in the red, and short choice trials in the green channel. The resulting video reveals a separation in body position from the first few hundreds of milliseconds (Figure 4, Supplementary Movie 2).

The differences in behavioral trajectories leading to different categorizations of the same stimuli imply that it should be possible to predict choice from behavioral trajectories. To quantify this effect, we employed a metric commonly used in sensory neuroscience known as *choice probability* (Britten et al., 1996; Nienborg et al., 2012). Choice probability is defined as the degree to which fluctuations of a variable during repeated presentations of a stimulus are predictive of perceptual judgments about that stimulus. This metric is commonly applied to the firing of neurons in sensory brain areas in order to estimate their involvement in the formation of percepts. We extend its use to assess whether body trajectories carried information about unfolding perceptual judgments of time intervals.

**Figure 5:**
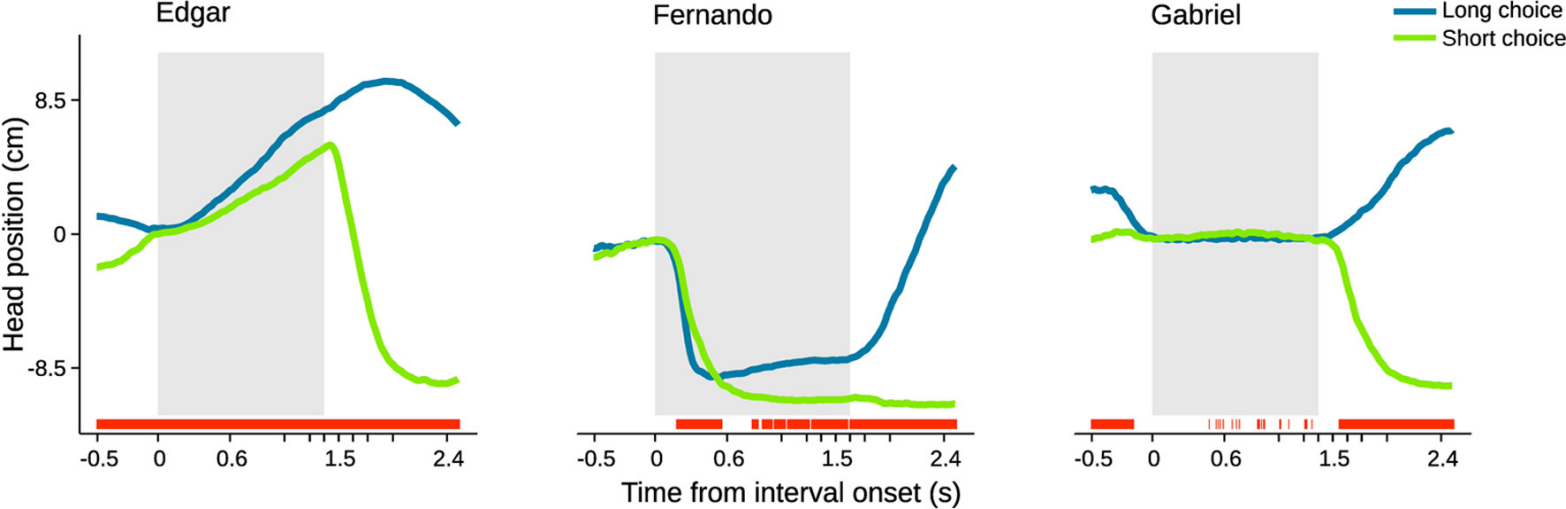
**Head trajectory is predictive of choice**. Average head trajectories leading to long (blue) and short (green) categorizations of same near boundary durations. Gray shaded area indicates stimulus interval period. For each subject, the stimulus of highest choice variance across sessions was selected. Red bars indicate moments when head position is significantly predictive of choice (95% bootstrap confidence intervals on auROC curve). *n* = {678, 475, 300} trials.

We started by calculating choice probability from head position at individual time steps within a period extending from 0.5 s before to 2.5 s after trial initiation (Figures 5, S2, S3). Choice probability from head position was quantified as the area under a receiver operating characteristic (ROC) curve. This curve was calculated using distributions of head position observed in short versus long choice trials (see Materials and Methods for details). In agreement with the example video, this analysis revealed that overt behavioral sequences often allow perceptual judgment to be predicted above chance. The profile of choice probability over time differed for each individual subject (Figure 5). Edgar displayed a monotonically increasing profile that is significant from before stimulus onset and throughout stimulus presentation. Fernando displayed a more complex profile that was significant early in the stimulus, lost significance, and then regained small but generally significant separability from 0.7 s onward. Gabriel did not display overt head trajectories during the interval period, staying at the initiation port throughout presentation of the stimulus interval instead. However, Gabriel's choice probabilities were significant prior to trial initiation. The absence of appreciable change in Gabriel's head position during the interval period made it impossible to extract any information from this variable. However, close inspection of individual videos suggested that this rat may have produced smaller scale movements around the initiation port in the axis normal to the image plane. We were not able to quantify such movements using the current setup, and thus likely underestimated the degree to which this animal's movement may have related to choice.

Our analysis of head position represents an instantaneous behavioral analog of neuronal choice probability. While these analyses provided a measure of how well choice can be predicted from head position at each point in time, it is possible that there was information about choice contained in head position over multiple time points.

**Figure 6:**
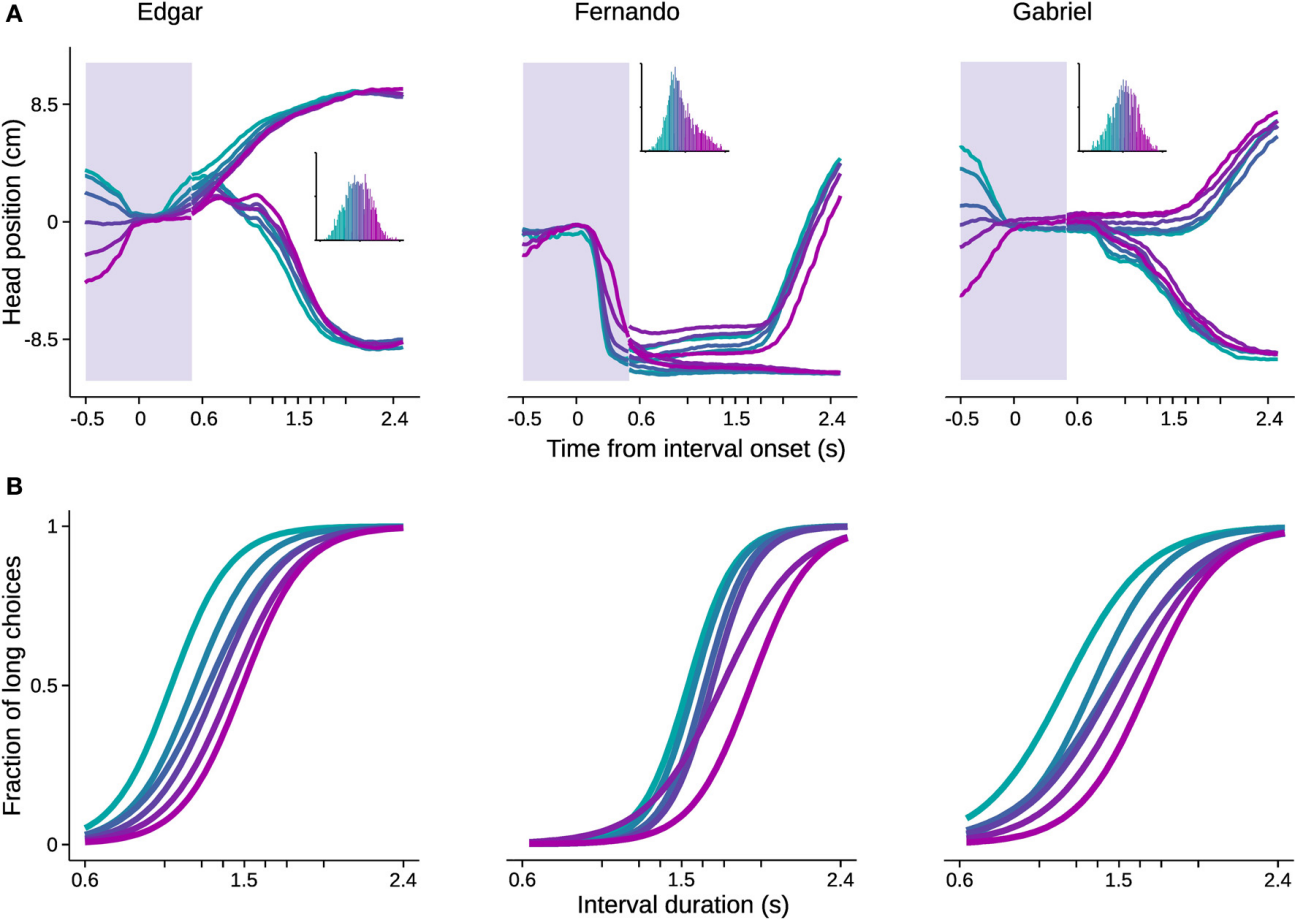
**Head trajectory reveals categorization bias**. Trajectories of head position in a 1 s time window centered on interval onset were used to predict choice. The probability of trajectories conditional on choice was fitted with Gaussian mixture models. The fitted distributions were used to estimate choice probability from single trial trajectories. Trials were then binned by choice probability. **(A)** Trajectories averaged within bins are shown for the time window used for prediction (shaded area). For the remaining time, mean trajectories were further split by choice. Color code indicates bins. Inset: histogram of choice probabilities estimated from trajectories. **(B)** Psychometric curves for trials comprised in each bin. *n* = {4542, 3680, 2641} trials. *n* = 6 bins.

In order to evaluate the impact of head trajectories on choice, we used standard methods of clustering in high dimensional space. Briefly, trajectories were represented as sequences of head positions during a time window extending from 0.5 s before trial initiation to 0.5 s after. Since the shortest possible stimulus duration is 0.6 s, this period is common across all trials, and the animal has not yet received any information about the stimulus duration being presented. Intuitively, if behavioral trajectories are systematically related to perceptual decisions, long and short choice trials should be distributed differently in high dimensional head position space. Choice probability was estimated from single trial trajectories by fitting the head position sequences with multivariate Gaussian mixture models (see Materials and Methods for details). Next, we grouped trials based on choice probability in six bins with equal number of trials, and plotted the mean trajectory and psychometric curve for each bin (Figure 6).

All psychometric curves asymptoted at 0 and 1, but biases were strikingly different and ordered. As noted, this analysis took as input behavior occurring before stimulus identity could possibly be known by the subject. Importantly, the ordered psychometric functions do not suggest that the animal has already formed its perceptual judgment prior to stimulus presentation. Were this the case, performance should be at chance level. Rather, the animal's head trajectory exerted a bias on choice, as the difference in psychometric functions was mainly captured by the bias parameter. The same pattern would be expected if we could bin trials with respect to internal decision variables such as clock speed or decision criterion.

### 2.4. Behavioral trajectory improves choice prediction beyond trial history

The average head trajectories during the period preceding trial initiation were strikingly related to choice probability (Figure 6A). This suggests that the predictive power of trajectories reflected events preceding the current trial, such as choices made and rewards received on recent trials (e.g., Sugrue et al., 2004; Lau and Glimcher, 2005). To test whether behavioral trajectories significantly improved our ability to predict choice beyond the information provided by trial history, we fit four logistic regression models to the choice data that differed in the combination of predictor variables included. We allowed different combinations of subject, current trial stimulus, recent trial history of choices, stimuli and rewards, and head trajectory to be weighed in predicting choice (Table 1; see Materials and Methods and Table 2 for details).

**Table 1:** **Behavioral trajectories improve choice prediction beyond trial history**.

**Model no.**	**Predictor groups**	**Prediction success (%)**	**Deviance**	**BIC**	**AIC**
1	Subject, interval	83.8	7103	7157.9	7115
2	Subject, interval, trajectory	84.5	6611	6711.7	6633
3	Subject, interval, trial history	84.4	6944.9	7073.1	6972.9
4	Subject, interval, trial history, trajectory	85.0	6544.5	6700.1	6578.5

Outcome of multivariate logistic regression models fit to predict choice from stimulus, trial history, and behavioral trajectory.

**Table 2:** **Specification of logistic regression models used to predict choice**.

**Model no.**	**Initial specification**	**Final specification**
1	*S* + *I*	*S* × *I*
2	*S* + *I* + *T*_PC1_ + *T*_PC2_	*S* × *I* + *T*_PC1_ + *S* × *T*_PC2_ + *I* × *T*_PC2_
3	*S* + *I* + *R*_*t*−1_ + *d*(*I*_*t*−1_) + *I*_*t*−1_	*S* × *I* + *R*_*t*−1_
4	*S* + *I* + *T*_PC1_ + *T*_PC2_ + *R*_*t*−1_ + *d*(*I*_*t*−1_) + *I*_*t*−1_	*S* × *I* + *T*_PC1_ + *S* × *T*_PC2_ + *I* × *T*_PC2_ + *R*_*t*−1_

The four models were initially specified will all linear terms and no interactions. Models were then modified by a stepwise procedure that added interactions or removed terms so as to minimize BIC. *S* = subject; *I* = interval; *T*_PC1_ and *T*_PC2_ = trajectory of head position on a one second time window centered on interval onset, projected on the first and second principal components calculated across trials; *R*_t−1_ = reward on previous trial; *d*(I_t−1_) = difficulty of the stimulus presented on previous trial, defined as the unsigned distance from the 1.5 s boundary. Models described following notation by Wilkinson and Rogers (1973).

Model no.1 captured the effect of stimuli, expected to be strong if animals learned the duration discrimination rule inherent in the task. Dummy variables standing for individual subjects were used to capture cross subject differences in psychometric functions. As expected, model no.1 predicted choice at a high success rate (Table 1), and was strongly significant as compared to a constant model (log likelihood ratio test, χ^2^ = 6.01 × 10^3^, *df* = 5, *p* « 0.01).

Next we assessed the contribution of behavioral trajectories during the time window used to calculate choice probability (Figure 6). Model no.2 maintained the predictors present in model no.1, to which it added two variables describing behavioral trajectories (i.e., projections on the first and second principal components; see Materials and Methods for details). This model showed a modest improvement in prediction success rate over model no.1. Better predictions are expected from models with more free parameters. We therefore employed formal model comparison methods by calculating Bayesian and Akaike information criteria (BIC and AIC, respectively). In summary, these methods impose a cost for adding free parameters. The improvement achieved by adding extra free parameters for trajectories outweighed the costs imposed by both methods (Table 1). In addition, a log likelihood ratio test indicated that the improvement of model no.2 over model no.1 is highly significant (χ^2^ = 492, *df* = 5, *p* « 0.01).

In order to assess the contribution of information regarding trial history, model no.3 added to predictors in model no.1 variables describing stimulus, difficulty and reward on the preceding trial. Similar results were obtained for model no.3 as for model no.2, both compared to model no.1 (Table 1; log likelihood ratio test, χ^2^ = 135, *df* = 1, *p* « 0.01). A direct comparison between BIC and AIC values of models 2 and 3 indicated that trajectories were in fact slightly more informative than trial history. Furthermore, neither of the simpler models were better than model no.4, a full model incorporating variables relative to subject, stimulus interval, trajectories and trial history (Table 1; log likelihood ratio tests relative to model no.4; model no.2: χ^2^ = 46, *df* = 1, *p* « 0.01; model no.3: χ^2^ = 403, *df* = 5, *p* « 0.01).

## 3. Discussion

Under environments with strong temporal regularities, animal behavior is known to become temporally structured (e.g., Skinner, 1948; Hodos et al., 1962; Anderson and Shettleworth, 1977; Haight and Killeen, 1991; Machado and Keen, 2003; Balcı et al., 2008; Ölveczky, 2011). Stemming from this observation, trajectories through behavioral states have been proposed to implement interval timing (Killeen and Fetterman, 1988; Machado, 1997). A prediction implied by this rationale is that trial to trial variations in the flow of behavioral sequences should correlate with trial to trial variations in temporal estimation. Consistent with this prediction, we found that behavioral trajectories differed between cases where the same intervals were categorized differently. Due to these correlated differences, choice could be significantly predicted from ongoing behavior.

While some sequential state timing models map states directly onto behaviors, many models posit a more abstract sequential state representation of time. These include neural network models that produce dynamics in response to input (Buonomano and Merzenich, 1995), successively broadening temporal basis functions for learning prediction via temporal difference or other learning rules (Grossberg and Schmajuk, 1989; Suri and Schultz, 1999; Ludvig et al., 2008), or models that time intervals through specific phase relationships amongst a diversity of oscillatory processes (Miall, 1989; Matell and Meck, 2000, 2004). Our data provides evidence in favor of state transition timing models, but it does not speak to the underlying neural mechanisms, and thus is consistent with all of the above sequential state models for timing.

Furthermore, our findings do not rule out the existence of dedicated timing mechanisms such as the pacemaker-accumulator model contained within scalar expectancy theory (Gibbon, 1977) or a sequential state timing mechanism wherein states do not map directly to behaviors. In other words the quantitative relationship between continuous behavior and subsequent choice we observed does not prove that behavior directly drives the perceptual process of time estimation. Behavior may instead simply reflect a more centrally mediated timing process. Alternatively, the rodents in our study may have indeed used behavioral sequences to estimate time in our task, with such a strategy being a useful but non-unique solution to the problem of how to estimate duration. Hence, our results alone merely suggest embodied strategies as one of a number of possible solutions to timing. Lastly, the brain is presumably responsible for the production of stereotyped motor sequences, and so in a trivial sense, the brain must be part of the system that estimates duration, even if this computation is to some degree dependent on ongoing behavior. Why might organisms include ongoing behavior in the process of timing? Limbs and muscles have mass and inertia, which may increase time constants present in movement to a degree that is harder to achieve within the nervous system alone (cf. Vogels et al., 2005). Additional experiments that manipulate the environment dynamically would help reveal whether behavior is upstream of the perceptual process of time estimation.

A number of brain structures have been implicated in interval timing. These areas include parietal cortex (Janssen and Shadlen, 2005), prefrontal cortex (Fuster, 2001; Kim et al., 2013), the basal ganglia (Maricq and Church, 1983; Matell and Meck, 2000, 2004; Fiorillo et al., 2003; Meck et al., 2008; Jin et al., 2009; Adler et al., 2012), and the cerebellum (Ivry and Keele, 1989). Interestingly from the perspective of embodied solutions to timing, these areas represent some of the most highly integrative territories in the mammalian brain, processing information concerning a broad range of sensory modalities and effector systems.

As is often the case, the full nature of interval timing likely reflects a mixture of various mechanisms, embodied and non-embodied alike (Wittmann, 2013). Animals and brains have evolved to be opportunistic, and are capable of employing a variety of strategies to solve cognitive tasks depending on the scenario they find themselves. The method of computing choice probabilities from behavior presented here represents a path forward for neuroscientists seeking to disambiguate embodied versus non-embodied components of cognitive acts such as perceptual decision making. In other decision-making contexts, it has been shown that information about an unfolding decision continually flows to the motor system, such that continuously observing behavioral output provides information about the dynamics of a decision process (Selen et al., 2012). In this way, ongoing behavior can provide a readout of the current state of decisions, which can then be compared to neural signals that are thought to be involved. We suspect that, whether studies involve human, non-human primate, rodent, or other species as experimental subjects, some neural correlates of decision variables are explained by changing motor output or sensory input resulting from ongoing behavior. We also suspect that some neural correlates of decision variables precede the emergence of decision variables in ongoing behavior, and we propose to focus on neural correlates of decision variables that meet this criterion. To do so would surely provide a better handle on the genesis of choice.

## 4. Materials and methods

All experiments were approved by the Champalimaud Foundation Bioethics Committee and the Portuguese Direção Geral Veterinária.

### 4.1. Duration categorization task

Three adult male wild type Long-Evans hooded rats and one adult male PV-Cre Black 6 mouse were trained to categorize time intervals as either long or short by making left/right choices. Animals learned to trigger stimulus intervals by nosepoking at the centrally located initiation nose port when it was illuminated. Triggering a stimulus would immediately turn off the initiation port light, and cause a pair of audible tones to be played separated in time by an interval randomly selected from the set *I* = {0.6, 1.05, 1.26, 1.38, 1.62, 1.74, 1.95, 2.4} s. Tones consisted of 150 ms long trains of square pulses at 7 kHz. Intervals in the set are symmetric around the 1.5 s categorical boundary, and make up four difficulty levels in geometric progression. The duration of the presented interval governed reinforcement of nosepoking at the laterally located choice ports, and responses were interpreted as the animal's perceptual judgment regarding interval duration. For intervals longer than the categorical boundary, a water reward became available for delivery upon choice of the left nose port, or at the right nose port for intervals shorter than the boundary. Incorrect responses were cued with a 150 ms long white noise sound and punished with an 11 s time out.

Animals were required to withhold poking at the choice ports during stimulus presentation, but were otherwise unrestrained. Responses occurring before interval offset were termed premature, and had the same consequences as incorrect choices (i.e., error tone and time out). Premature responses occurred in 6.7 ± 3.3% and 21.3 ± 5.6% of trials (rats and mouse, respectively; mean ± standard deviation). Stimulus intervals interrupted by premature responses were repeated in the subsequent trials, i.e., animals could not skip long intervals by making premature responses.

Nine seconds after the initiation of the previous trial, or twenty seconds following incorrect/premature responses, the initiation port would become illuminated again, indicating that a new trial could be initiated.

Sessions were selected based on categorization performance. In the selected sessions, animals correctly categorized the easiest stimuli (i.e., 0.6 and 2.4 s) at a rate of 95.8 ± 0.03%, while performance reached 69.4 ± 5.58% for the hardest stimuli (i.e., 1.38 and 1.62 s), and 84.7 ± 3.57% over all eight stimuli (mean ± standard deviation). Psychometric functions are presented as logistic regressions fit to predict probability of a long choice from the duration of presented stimulus intervals. Logistic regressions were fit to single sessions. Performance of individual subjects was summarized by averaging over the parameters fit to single sessions.

### 4.2. Behavioral set up

Behavioral boxes consisted of a metal cage (mice, Island Motion, Tappan, NY, USA) or a plastic bucket (rats, IKEA, Alfragide, Portugal) containing one speaker (Cover Industrial Co., Guangdong, China) and three nose ports (Island Motion). Each nose port contained one infra-red beam/sensor pair for detecting nosepoking and one visible LED. The choice ports contained, in addition, a water tube connected to a solenoid valve for reward delivery. Valves were calibrated to deliver 25 or 5 μl of water per reward event (rats and mouse, respectively).

Except for the video camera, all sensors and effectors in the behavioral box were read and controlled by an Arduino Mega 2560 microprocessor (additional information and free software available at http://www.arduino.cc/) via a custom circuit board. The microprocessor implemented the behavioral task, and, through a serial communication port, outputted data to a desktop computer running custom software based on Python's pySerial module (freely available at http://pyserial.sourceforge.net/).

### 4.3. Video acquisition and tracking

Video was acquired with a high speed camera (Flea3 FL3-U3-13S2C-CS, Point Grey Research Inc., Richmond, Canada) at 120 frames/s with a resolution of 1280 × 960 pixels in grayscale at 8 bits (rats), or 90 frames/s at 640 × 480 pixels (mouse). Video acquisition and offline tracking were performed using the in-house developed software Bonsai (freely available at http://bitbucket.org/horizongir/bonsai/downloads). To extract position of the head from the raw videos, images were background subtracted and thresholded so that the animal's body appeared as a distinct blob. For each frame, the blob's largest axis was found, and the spatial position of the axis tip corresponding to the animal's head was tracked in both x and y dimensions. All analyses were carried on position along the axis in which nose ports are lined up, while motion along the orthogonal axis was discarded.

### 4.4. Estimating choice probability from ongoing behavior

#### 4.4.1. Momentary head position

Choice probability given head position at a particular time, *P*(*C*|*H*_*t*_), was calculated by applying an ROC analysis (Green and Swets, 1966) to assess the degree of overlap between the two known distributions *P*(*H_t_*|*C*) for *C* ϵ {long, short}. The area under the ROC curve was calculated and rectified about 0.5. This number signifies the probability that an ideal observer (i.e., one with full knowledge of the distributions) would correctly categorize a new sample. Effectively, it provides an instantaneous metric of the degree to which head position is informative about unfolding perceptual decisions. A 95% confidence interval around chance level was estimated by calculating choice probability over randomly shuffled data. The procedure was repeated 100 times for each time step, and the 95th percentile was taken as the confidence interval. The analysis was applied separately for each subject and stimulus interval.

#### 4.4.2. Behavioral trajectories

Choice probability was also estimated from behavioral trajectories. Trajectories were defined as vectors of head positions during a one second long time window centered on interval onset, and are denoted by **H**. This time window was chosen because during this period subjects had no information about the stimulus being presented, thus allowing us to combine trials of different stimulus types in the same analysis. We proceeded by fitting Gaussian mixture models to the two known multivariate distributions *P*(**H**|*C*) for *C* ϵ {long, short}. On the fitting procedure, trajectories were weighted by choice variance. The rationale is that stimulus is a near-sufficient predictor of choice variability for easy stimuli, as made clear by the psychometric curves. In other words, variability emerging from other sources, such as body dynamics, would only impact decision to the extent allowed by stimulus ambiguity. Therefore, in order to capture the relevant variability, trajectories occurring in trials of a given session and stimulus type contributed to fitting with weights given by the associated binomial variance of choice. Once the distributions *P*(**H**|*C*) were fitted, and given knowledge of the marginal distributions *P*(*C*) and *P*(**H**), we could use Bayes theorem to calculate the choice probability *P*(*C*|**H**) for individual trials as follows:

(1) $$P(C|H)=\frac{P(H|C)\times P(C)}{P(H)}$$

Trials were binned based on choice probability to generate the trajectories and psychometric functions presented in Figure 6. Similar results were observed when trajectories were fit using flat weights (data not shown). The analysis was applied separately for each subject.

### 4.5. Generalized linear models

We fitted four logistic regression models to choice data. The models included different combinations of predictor variables referring to subject, stimulus, behavioral trajectory and trial history (Table 1). Subjects were represented by categorical variables, while stimulus duration was represented as a continuous variable. Behavioral trajectories were considered within the same time window used to calculate choice probability (i.e., a 1 s time window centered on interval onset). Given the rate at which videos were acquired, trajectory excerpts of this length are variables of 120 dimensions. To avoid adding unnecessary free parameters to the logistic models, a principal component analysis was run across the whole dataset. The first two principal components explained 89.8% of trajectory variance, and single trial projections onto these were fed to the logistic model. Trial history was represented by three variables referring to trials prior to choice: stimulus duration, stimulus difficulty (defined as the unsigned distance from the 1.5 s categorical boundary), and reward (defined as 1 for reward after long choices, 0 for no reward, and −1 for reward after short choices). Initially, models were specified with all linear terms and no interactions (Table 2, middle column). Models were then modified by a stepwise procedure that added interactions or removed terms so as to minimize BIC. Final model specifications are shown in the last column of Table 2. This analysis did not include trials in which a premature response occurred, nor their immediately succeeding trials.

### Conflict of interest statement

The authors declare that the research was conducted in the absence of any commercial or financial relationships that could be construed as a potential conflict of interest.

## Supplementary material

Supplementary Movie 1 **Average video of representative session by Fernando depicting trials in which stimulus interval was the longest, 2.4 s**. In the depicted session, he correctly chose the long choice port for all presentations of this stimulus.

Supplementary Movie 2 **Average video of representative session by Edgar depicting trials of stimulus interval 1.38 s, the one for which choice variability was highest**. Trials in which he correctly judged the interval as short are in the green color channel. Trials in which he mistakenly judged the interval as long are in the red color channel.

Figure S1 **Correlation between behavioral trajectory and temporal categorization was replicated in the mouse**. **(A)** Psychometric function shows near perfect categorization of easiest stimuli, while performance approaches chance level for intermediate, near boundary intervals. Thin lines are logistic fits to single sessions. Thick line summarizes performance across sessions. *n* = 6 sessions. 201 ≤ *n* ≤ 521 trials per session. **(B)** Average head trajectories around presentations of the longest interval. Thin lines are single session means. Thick line is mean of session means. *n* = 109 trials. Gray shaded area indicates stimulus interval period. **(C)** Average head trajectories leading to long (blue) and short (green) categorizations of a near boundary stimulus interval. Red bar indicates moments when head position is significantly predictive of choice (95% bootstrap confidence intervals). *n* = 553 trials. **(D)** Choice probability was estimated from head trajectory around stimulus onset time (purple shaded area), and trials were then binned by choice probability. Trajectories averaged within bins are shown for the time window used for prediction. For the remaining time, mean trajectories were further split by choice. Color code indicates bins. Inset: histogram of choice probabilities. **(E)** Psychometric curves for trials comprised in each bin. *n* = 2137 trials. *n* = 6 bins.

Figure S2 **Head trajectories leading to different categorizations of each of the eight stimulus durations**. Blue and green curves depict average head trajectories leading to long and short categorizations, respectively. Stimulus period is indicated by gray shaded area. Red asterisks indicate panels presented in Figure 5 (rats) and Figure S1 (mouse). Mouse BII made no incorrect choices after presentations of the shortest interval.

Figure S3 **Choice probability given momentary head position**. Choice probability calculated from momentary head position. Stimulus period is indicated by the gray shaded areas. Colored shaded areas indicate 95% bootstrap confidence intervals around chance. For mouse BII, analysis could not be applied to shortest interval due to absence of incorrect choices.
