# Supplementary figures and images for: Ongoing behavior predicts perceptual report of interval duration
Source: Front Neurorobot. 2014 Mar 11;8:10. doi: 10.3389/fnbot.2014.00010 (PMC3949350; doi:10.3389/fnbot.2014.00010)

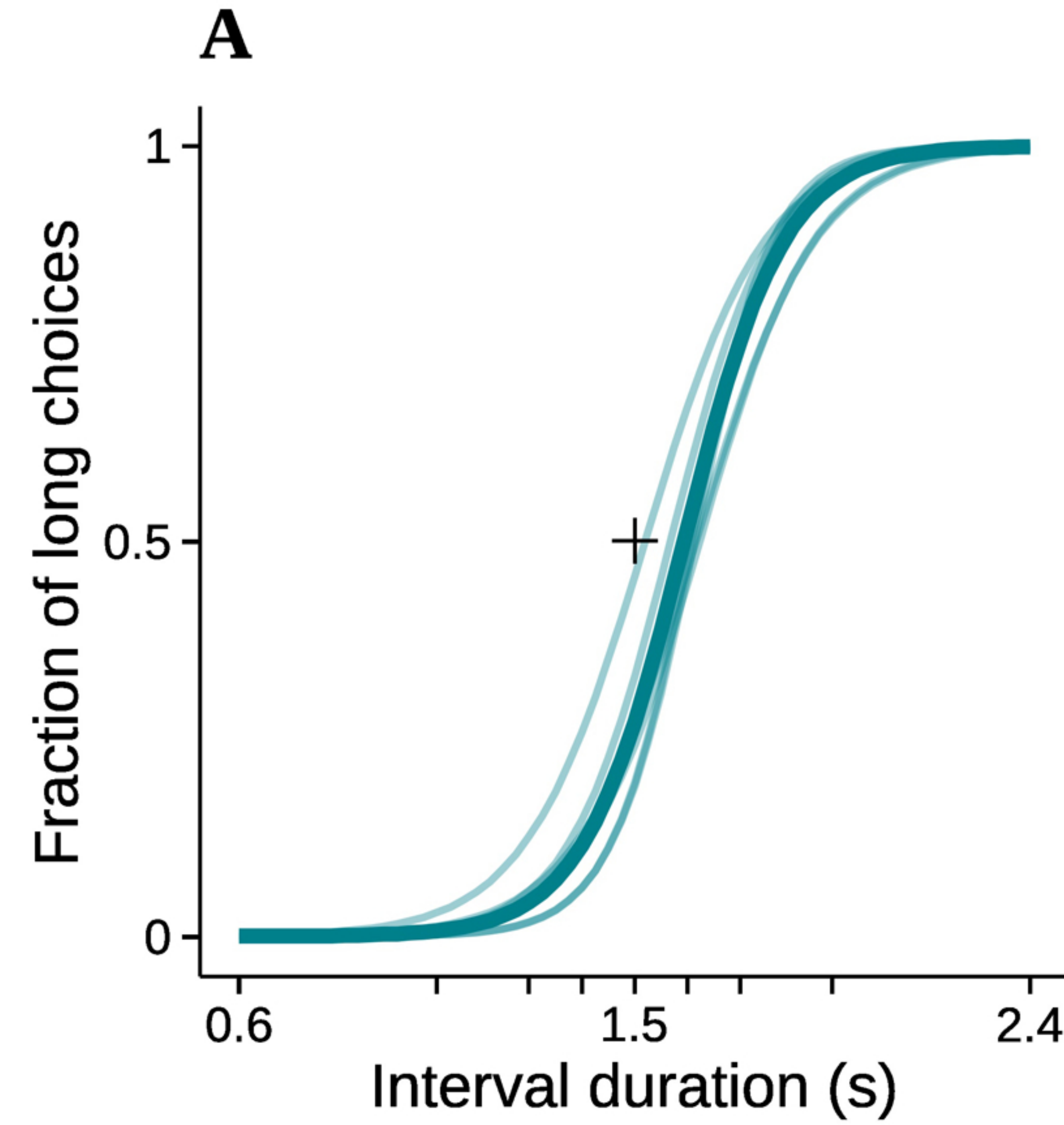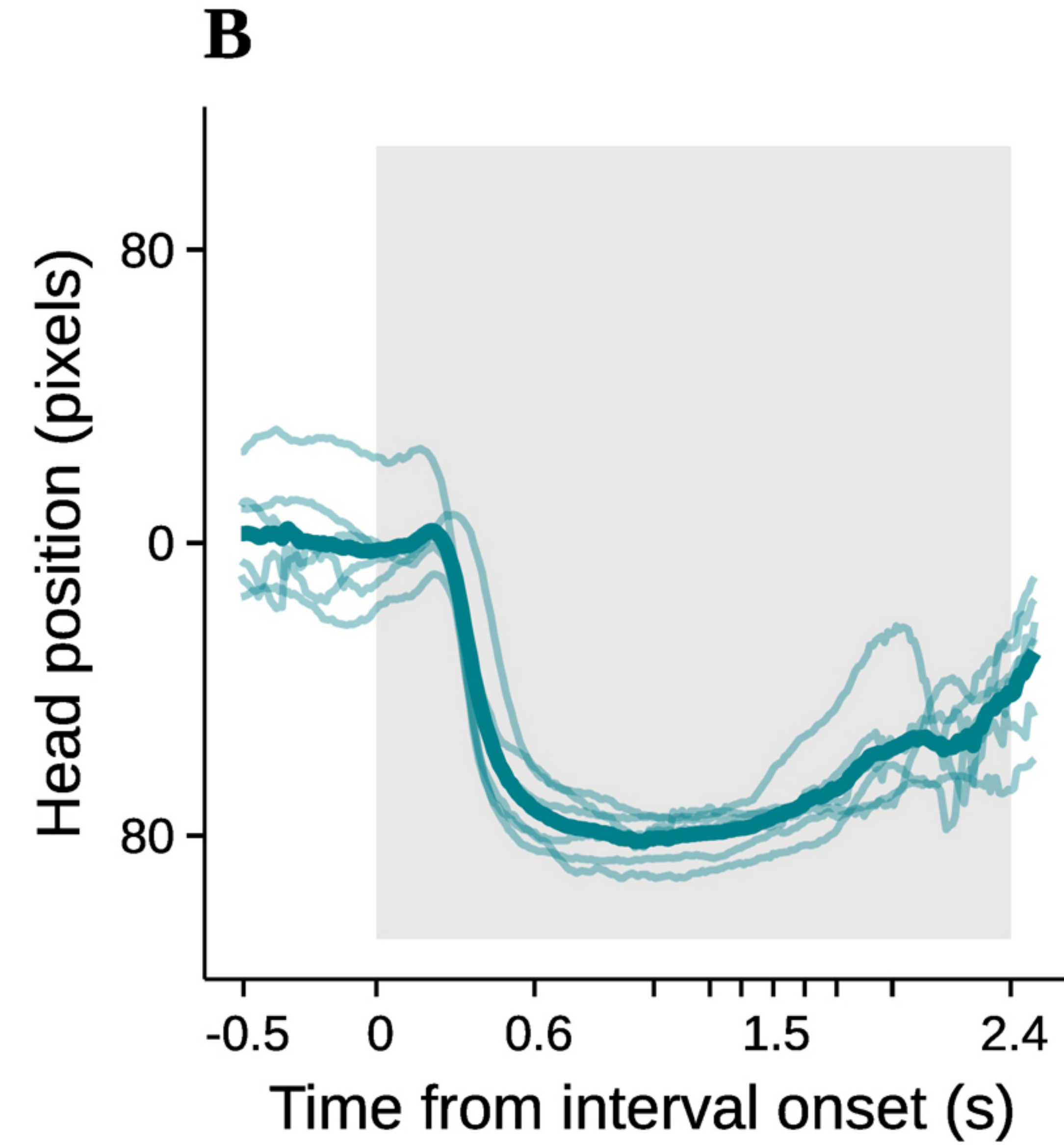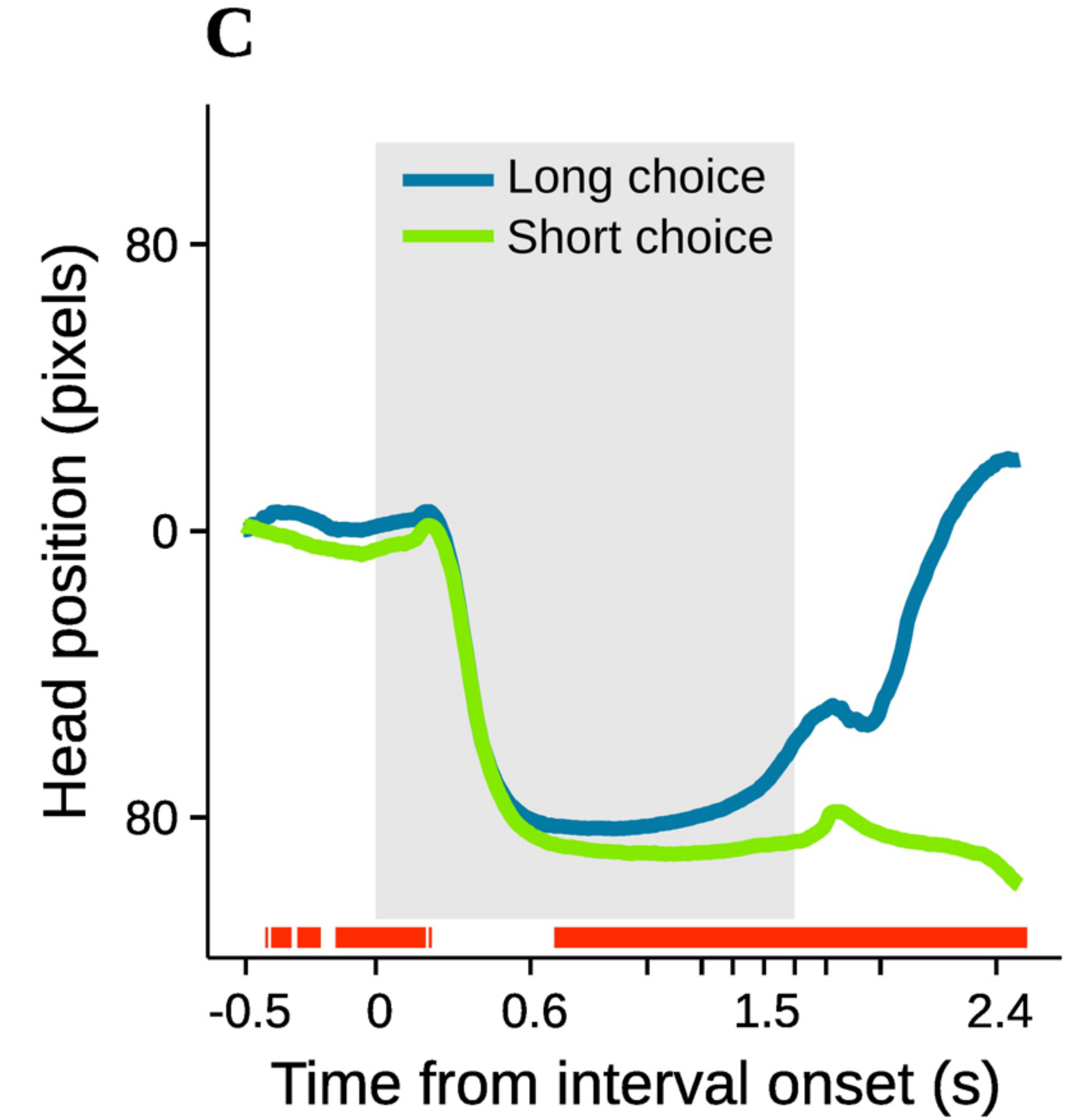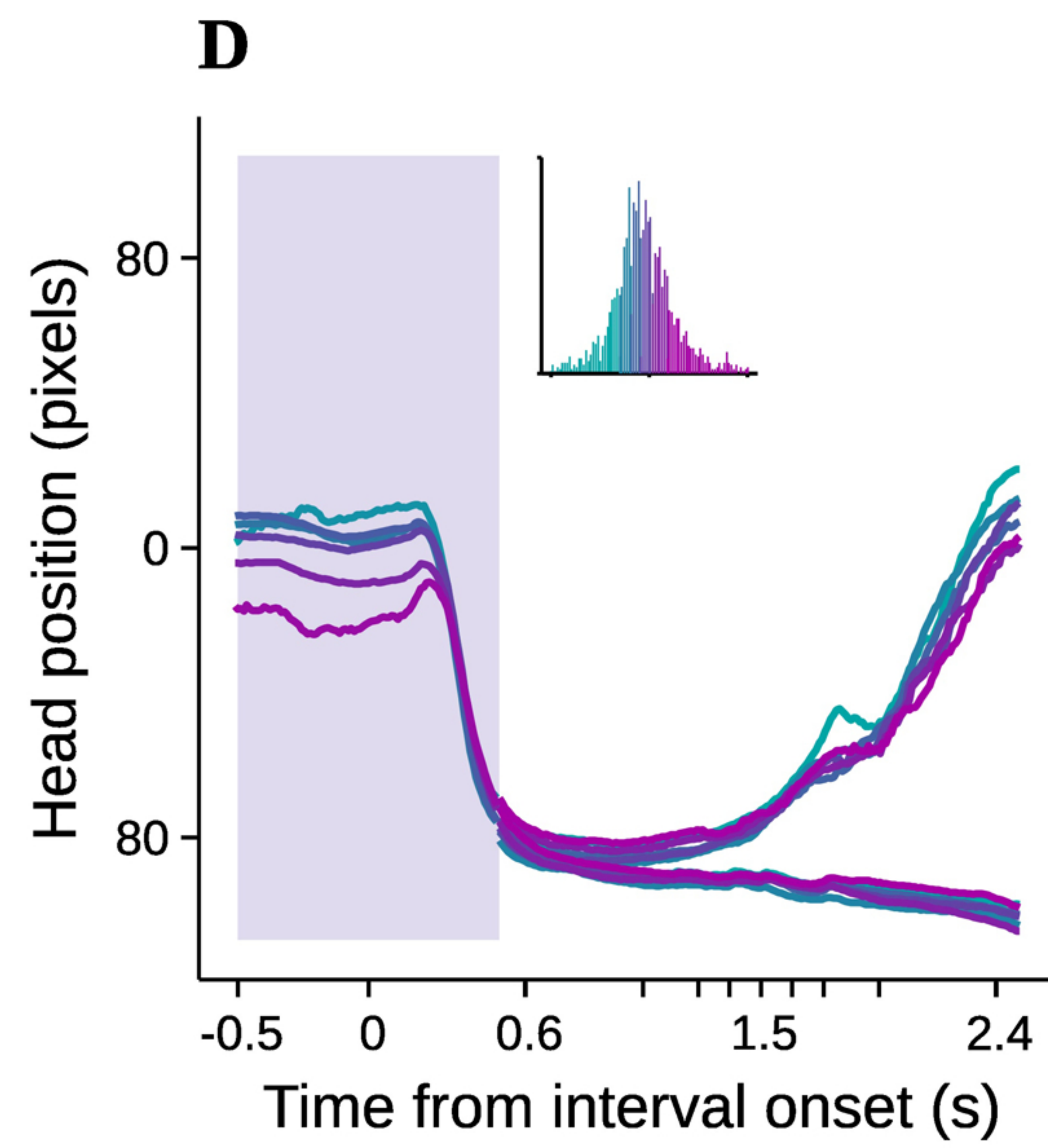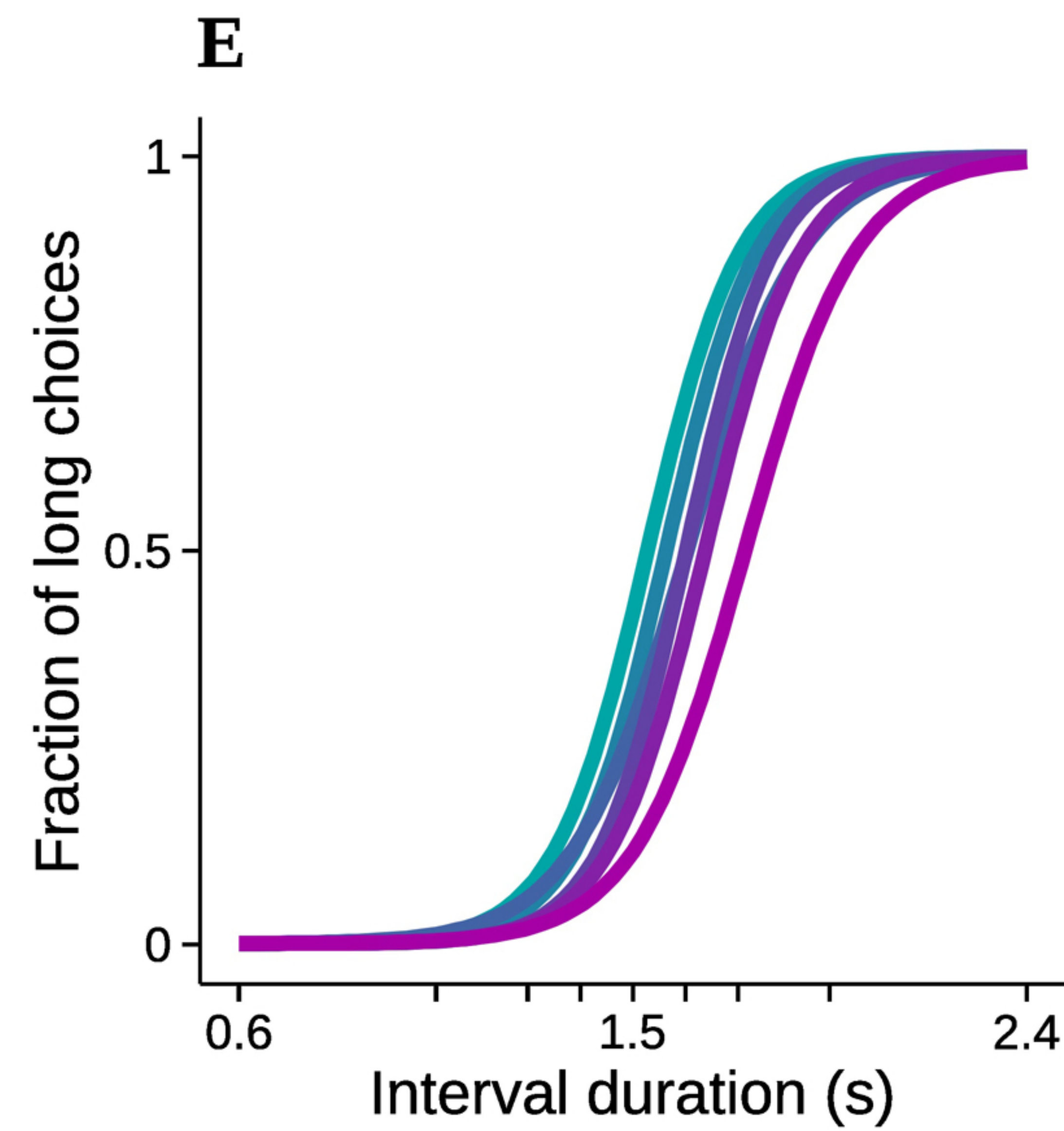

Supplement: Figure S1 — Correlation between behavioral trajectory and temporal categorization was replicated in the mouse. (A) Psychometric function shows near perfect categorization of easiest stimuli, while performance approaches chance level for intermediate, near boundary intervals. Thin lines are logistic fits to single sessions. Thick line summarizes performance across sessions. n = 6 sessions. 201 ≤ n ≤ 521 trials per session. (B) Average head trajectories around presentations of the longest interval. Thin lines are single session means. Thick line is mean of session means. n = 109 trials. Gray shaded area indicates stimulus interval period. (C) Average head trajectories leading to long (blue) and short (green) categorizations of a near boundary stimulus interval. Red bar indicates moments when head position is significantly predictive of choice (95% bootstrap confidence intervals). n = 553 trials. (D) Choice probability was estimated from head trajectory around stimulus onset time (purple shaded area), and trials were then binned by choice probability. Trajectories averaged within bins are shown for the time window used for prediction. For the remaining time, mean trajectories were further split by choice. Color code indicates bins. Inset: histogram of choice probabilities. (E) Psychometric curves for trials comprised in each bin. n = 2137 trials. n = 6 bins. [file Presentation1.PDF]

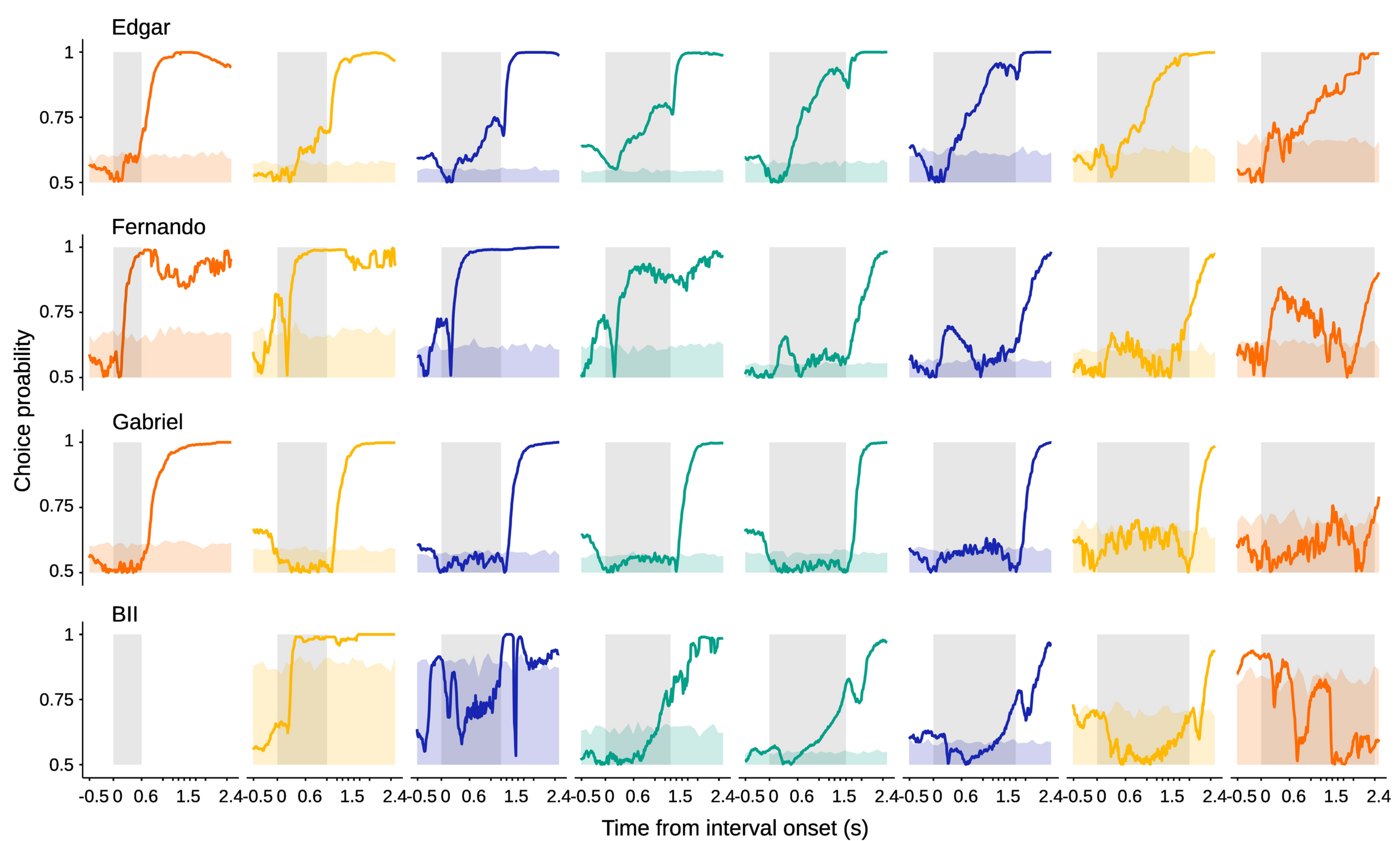

Supplement: Figure S2 — Head trajectories leading to different categorizations of each of the eight stimulus durations. Blue and green curves depict average head trajectories leading to long and short categorizations, respectively. Stimulus period is indicated by gray shaded area. Red asterisks indicate panels presented in Figure 5 (rats) and Figure S1 (mouse). Mouse BII made no incorrect choices after presentations of the shortest interval. [file Presentation2.PDF]
